# The Royal British Nurses' Association and the Nurse Training Schools

**Published:** 1891-05-02

**Authors:** Rutherford Alcock, Harry Verney, J. G. Wainwright, E. H. Lushington, E. Murray Ind, Henry Wace, W. Rathbone

**Affiliations:** President of the Metropolitan and National Association and Westminster Hospital and Training School; Vice-President of Westminster Hospital and Training School; Chairman of the Council of the Night- ingale Fund; Treasurer of St. Thomas's Hospital; Treasurer of Guy's Hospital; Chairman of the London Hospital; Chairman of the Committee of Manage- ment of King's College Hospital; President of the Liverpool Training School and Home for Nurses


### The Royal British Nurses' Association and the Nurse Training Schools.

To the Editor of The Hospital.

Sir,—At a meeting of the Royal British Nurses' Association, held on the 16th of this month, the proceedings of which have been reported in several of the daily papers, more than one of the principal speakers, including the Chairman and Hon. Secretary, spoke in strong terms of the persecution which, as they allege, this Association suffered at the hands of the nurse training schools and their representatives, and complained that this opposition aimed, amongst other things, at rendering the presidency of her Royal Highness the Princess Christian impossible, and at preventing Lady Bloomfield's Pension Fund for Nurses being handed over toit.

It would be making too great a demand on your space to ask the use of your columns, and at the present moment this seems hardly necessary, to discuss at length the grounds of the opposition of the nurse training schools and large hospitals to that which is the main object of the Royal British Nurses' Association, viz., the formation and control of a general register of nurses having an authoritative character—or to dwell upon the evil, both to the public and to nursing, which they foresee as the consequence, inasmuch as in all probability the matters in dispute will have to be fought out before the President of the Board of Trade.

But meanwhile we, the representatives of the leading nurse-training schools, feel it our duty to protest against the charge of using "secret means," and the term "persecution" being applied to the action of the bodies we represent. It is to the nurse training schools that the great advance which has taken place during the past thirty years in the quality of nurses and nursing and in the position of nurses is wholly due, and it is to these that we must look in the future for the maintenance of the high standard of excellence which has been thus attained.

Can it be seriously, then, charged against the authorities of these schools, all of whom have worked in the interest of nurses and of nursing—and many have devoted themselves to the subject for years, and are themselves trained nurses—that they are the persecutors of nurses, or that they are perversely opposing schemes which are "obviously for the advantage of the nursing profession"?

They do not oppose, and never have opposed, the formation of any institution established for the purpose of thrift, mutual benefit, or of social intercourse; but believing as they do, for reasons which they have elsewhere fully explained, that the establishment of such a register, and the granting by such a body as the Royal British Nursing Association of certificates of competency to nurses, will be detrimental to the interest of nurses and misleading to the public and medical profession, they certainly have opposed, and will continue to oppose, the attempt of the Association to obtain from the Board of Trade the privilege which it seeks, and by which a "legal status" would be accorded toit.

With regard to the Lady Bloomfield Fund, not one single word has been said by the nurse training schools or hospitals on the subject. The Lady Bloomfield Fund has a most distinct and valuable object in view, and with her Ladyship as its head, and under the guidance of his Grace the Duke of Westminster, will without doubt be most successfully and carefully managed. Whether its transference to any other association would be wise or not can only have been brought into the discussion for the purpose of diverting attention from the real question at issue.

While recognising in full the great interest taken by her Royal Highness the Princess Christian in the welfare of nurses and its value to nurses, they cannot but regard the manner in which her Royal Highness's name has been introduced into the discussion as wholly uncalled for and unworthy of the spirit of fair discussion.

Westminster, President of the Metropolitan and National Association and Westminster Hospital and Training School.

Rutherford Alcock, Vice-President of Westminster Hospital and Training School.

Harry Verney, Chairman of the Council of the Nightingale Fund.

J. G. Wainwright, Treasurer of St. Thomas's Hospital.

E. H. Lushington, Treasurer of Guy's Hospital.

E. Murray Ind, Chairman of the London Hospital.

Henry Wace, Chairman of the Committee of Management of King's College Hospital.

W. Rathbone, President of the Liverpool Training School and Home for Nurses.

